# Relebactam restores susceptibility of resistant *Pseudomonas aeruginosa* and Enterobacterales and enhances imipenem activity against chromosomal AmpC-producing species: analysis of global SMART 2018–2020

**DOI:** 10.1186/s12866-023-02864-3

**Published:** 2023-06-13

**Authors:** David W. Hilbert, C. Andrew DeRyke, Mary Motyl, Meredith Hackel, Katherine Young

**Affiliations:** 1 Merck & Co., Inc, Rahway, NJ USA; 2 International Health Management Associates, Inc, Schaumburg, IL USA

**Keywords:** Minimum inhibitory concentration, Gram-negative bacteria, Antibacterial agents, Bacterial infections, Antibacterial susceptibility, Imipenem, Relebactam, Carbapenem

## Abstract

**Background:**

Carbapenem-resistant bacteria are an increasing problem in clinical practice; thus, it is important to identify β-lactamase inhibitors (*e.g.*, relebactam) that can restore carbapenem susceptibility. We report analyses of relebactam enhancement of imipenem activity against both imipenem-nonsusceptible (NS) and imipenem-susceptible (S) *Pseudomonas aeruginosa* and Enterobacterales. Gram-negative bacterial isolates were collected for the ongoing Study for Monitoring Antimicrobial Resistance Trends global surveillance program. Clinical and Laboratory Standards Institute–defined broth microdilution minimum inhibitory concentrations (MIC) were used to determine the imipenem and imipenem/relebactam antibacterial susceptibilities of *P. aeruginosa* and Enterobacterales isolates.

**Results:**

Between 2018 and 2020, 36.2% of *P. aeruginosa* (*N* = 23,073) and 8.2% of Enterobacterales (*N* = 91,769) isolates were imipenem-NS. Relebactam restored imipenem susceptibility in 64.1% and 49.4% of imipenem-NS *P. aeruginosa* and Enterobacterales isolates, respectively. Restoration of susceptibility was largely observed among *K. pneumoniae* carbapenemase-producing Enterobacterales and carbapenemase-negative *P. aeruginosa*. Relebactam also caused a lowering of imipenem MIC among imipenem-S *P. aeruginosa* and Enterobacterales isolates from chromosomal Ambler class C β-lactamase (AmpC)–producing species. For both imipenem-NS and imipenem-S *P. aeruginosa* isolates, relebactam reduced the imipenem MIC mode from 16 μg/mL to 1 μg/mL and from 2 μg/mL to 0.5 μg/mL, respectively, compared with imipenem alone.

**Conclusions:**

Relebactam restored imipenem susceptibility among nonsusceptible isolates of *P. aeruginosa* and Enterobacterales and enhanced imipenem susceptibility among susceptible isolates of *P. aeruginosa* and isolates from Enterobacterales species that can produce chromosomal AmpC. The reduced imipenem modal MIC values with relebactam may result in a higher probability of target attainment in patients.

**Supplementary Information:**

The online version contains supplementary material available at 10.1186/s12866-023-02864-3.

## Introduction

Carbapenems, members of the class of broad-spectrum antibacterial agents known as β-lactams, are an option for multidrug-resistant infections that may fail initial lines of therapy [1]. Nonsusceptibility to carbapenem treatment is frequently due to synergistic resistance mechanisms present within certain pathogenic strains (*e.g.*, concurrent porin loss, modification of penicillin-binding proteins, and/or expression of β-lactamases [expanded spectrum or Ambler class C (AmpC), including *Pseudomonas*-derived cephalosporinase (PDC)]) [2, 3]. AmpC can either be encoded by chromosomal genes, inducible upon exposure to certain β-lactam antibacterial agents, such as imipenem [4], or plasmid-acquired and generally constitutively expressed [5]. Chromosomal AmpC is a particularly important mechanism of resistance for numerous β-lactam antibacterial agents in the treatment of *P. aeruginosa* [6]. In addition, among Enterobacterales, certain species such as *Citrobacter freundii*, *Enterobacter cloacae*, *Klebsiella aerogenes,* and *Serratia marcescens* encode a chromosomal AmpC enzyme whose expression can be de-repressed, either by genetic mutation or the presence of an inducing β-lactam antibacterial, such as imipenem [6]. Some carbapenem-resistant Enterobacterales isolates do not produce a carbapenemase, and resistance is due to the presence of an extended spectrum β-lactamase or AmpC enzyme in combination with loss of expression of outer membrane porins. In addition, certain physiologic conditions among critically ill patients, such as augmented renal clearance (ARC), may lead to underdosing, which can contribute to inadequate response to therapy [7, 8]. Some β-lactams are particularly susceptible to subtherapeutic treatment exposures that may result from ARC, especially among isolates with minimum inhibitory concentrations (MIC) at the higher end of the susceptibility range, limiting their bactericidal activity [7, 8]. Development of suitable β-lactam/β-lactamase inhibitor combinations, such as imipenem/cilastatin/relebactam (IMI/REL), capable of overcoming loss of carbapenem susceptibility and limiting potential for underexposure, is important because of the ongoing global threat of multidrug-resistant bacteria and the potential for inadequate dosing in critically ill patients [7–13].

Relebactam is an inhibitor of Ambler class A and class C (*e.g.*, AmpC) β-lactamases that, when combined with imipenem, restores imipenem activity against nonsusceptible isolates and enhances imipenem activity, specifically against susceptible *P. aeruginosa* isolates [2]. In a fixed-dose combination with imipenem/cilastatin, relebactam was approved in the United States and European Union for hospital-acquired pneumonia and ventilator-associated pneumonia, bacteraemia associated with hospital-acquired pneumonia/ventilator-associated pneumonia (European Union only), and infections due to aerobic gram-negative organisms in adults with limited treatment options (*e.g.*, complicated urinary tract infections [cUTI] and complicated intra-abdominal infections [cIAI]) [14, 15].

Previous analysis of in vitro activity in imipenem-nonsusceptible (NS) isolates indicated that relebactam lowers MICs through inhibition of β-lactamase activity [2, 16]. Here, we expand upon previous reports of relebactam potentiation of imipenem activity against both imipenem-NS and imipenem-susceptible (S) *P. aeruginosa* surveillance isolates and extend this analysis to both imipenem-NS and imipenem-S Enterobacterales isolates from the ongoing Study for Monitoring Antimicrobial Resistance Trends (SMART) global surveillance program [16, 17]. The SMART program was initiated in 2002 and includes collection and assessment of clinical isolates by hospital laboratories for monitoring antibacterial susceptibility profiles of gram-negative bacteria [17].

## Results

Between 2018 and 2020, 23,073 *P. aeruginosa* isolates and 91,769 Enterobacterales isolates were collected from patients at sites participating in the SMART program. For *P. aeruginosa*, 36.2% (*n* = 8356) of isolates were classified as imipenem-NS according to the Clinical and Laboratory Standards Institute (CLSI) breakpoint (MIC > 2 μg/mL). For Enterobacterales, 8.2% (*n* = 7493) of isolates were classified as imipenem-NS according to the CLSI breakpoint (MIC > 1 μg/mL).

**Fig. 1:**
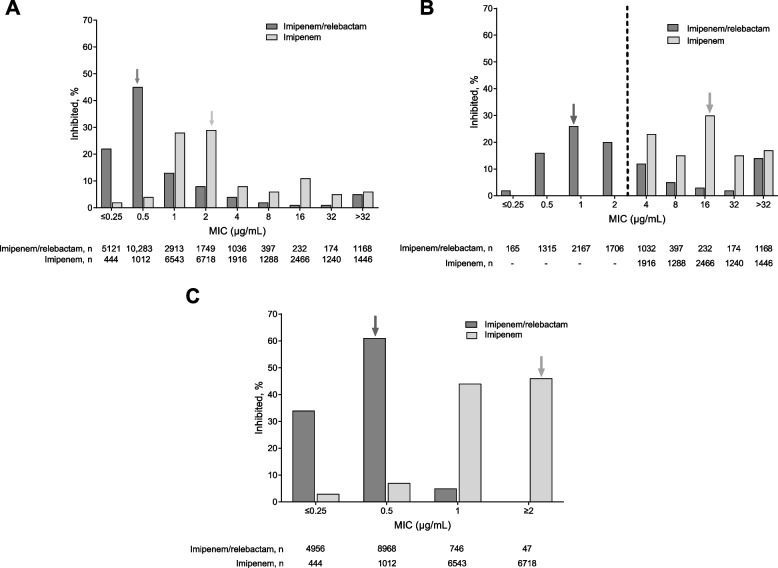
Relebactam restores/enhances the activity of imipenem in *Pseudomonas aeruginosa* isolates. **A** all (*N* = 23,073). **B** imipenem-NS (*N* = 8356). **C** imipenem-S (*N* = 14,717). Percentage represents *n*/*N* × 100%, where *n* was the number of isolates meeting the MIC threshold and *N* was the total number of isolates based on the CLSI 2021 clinical breakpoints for imipenem/relebactam (MIC ≤ 2 μg/mL for susceptibility) and subsequently categorized as either S (MIC ≤ 2 μg/mL) or NS (MIC > 2 μg/mL) [18]. The dashed line indicates the CLSI 2021 imipenem/relebactam susceptibility breakpoints. The arrows indicate mode MIC values. CLSI, Clinical and Laboratory Standards Institute; MIC, minimum inhibitory concentration; NS, nonsusceptible; S, susceptible

Among *P. aeruginosa* isolates (*N* = 23,073), the presence of relebactam increased imipenem susceptibility from 63.8% to 87.0% (Fig. 1A) and reduced the MIC_50/90_ from 2/32 to 0.5/4 µg/mL; the mode MIC was reduced from 2 to 0.5 µg/mL (Table 1). The addition of relebactam restored imipenem susceptibility to 5353 (64.1%) of 8356 imipenem-NS *P. aeruginosa* isolates (Fig. 1B), reduced the MIC_50_ from 16 to 2 µg/mL and the mode MIC from 16 to 1 µg/mL (Table 1). Among molecularly characterized isolates, carbapenemases were rarely identified (0.2%) in imipenem-NS isolates for which relebactam restored susceptibility, and metallo-β-lactamases (MBL) were the most common carbapenemase (37.3%) in imipenem-NS isolates for which relebactam did not restore imipenem susceptibility (see Additional file 1). In addition, relebactam enhanced imipenem susceptibility among isolates of *P. aeruginosa* classified as imipenem-S (*n* = 14,717) by causing a shift toward lower MIC values (Fig. 1C), with the MIC_50/90_ decreasing from 1/2 µg/mL for imipenem alone to 0.5/0.5 µg/mL for imipenem/relebactam; the mode MIC was reduced from 2 µg/mL for imipenem to 0.5 µg/mL for imipenem/relebactam (Table 1).

**Table 1:** MIC_50/90_ and mode MIC values for imipenem-S, imipenem-NS, and total *Pseudomonas aeruginosa* and Enterobacterales.^a^

Organism	Drug	All Isolates				CLSI								EUCAST							
						IMI-S Isolates				IMI-NS Isolates				IMI-S Isolates				IMI-NS Isolates			
		*N*	MIC_50_	MIC_90_	Mode MIC	*N*	MIC_50_	MIC_90_	Mode MIC	*N*	MIC_50_	MIC_90_	Mode MIC	*N*	MIC_50_	MIC_90_	Mode MIC	*N*	MIC_50_	MIC_90_	Mode MIC
*Pseudomonas aeruginosa*	IMI	23,073	**2**	**32**	**2**	14,717	**1**	**2**	**2**	8356	**16**	> 32	**16**	16,633	**2**	**4**	**2**	6440	**16**	> 32	**16**
	IMR		**0.5**	**4**	**0.5**		**0.5**	**0.5**	**0.5**		**2**	> 32	**1**		**0.5**	**1**	**0.5**		**2**	> 32	**1**
Enterobacterales	IMI	91,769	**0.25**	**1**	≤ 0.12	84,276	**0.25**	0.5	≤ 0.12	7493	** > 8**	> 8	> 8	86,178	**0.25**	0.5	≤ 0.12	5591	** > 8**	> 8	> 8
	IMR		** ≤ 0.12**	**0.5**	≤ 0.12		** ≤ 0.12**	0.5	≤ 0.12		**2**	> 8	> 8		** ≤ 0.12**	0.5	≤ 0.12		**4**	> 8	> 8
Chromosomal AmpC producers	IMI	13,003	**0.5**	**2**	**0.5**	11,369	**0.5**	**1**	**0.5**	1634	**2**	> 8	**2**	12,441	**0.5**	1	**0.5**	562	** > 8**	> 8	> 8
	IMR		**0.25**	**1**	**0.25**		**0.25**	**0.5**	**0.25**		**1**	> 8	**0.25**		**0.25**	1	**0.25**		**4**	> 8	> 8
*Enterobacter cloacae*	IMI	4784	**0.5**	**1**	**0.5**	4432	**0.5**	**1**	**0.5**	352	**8**	> 8	> 8	4561	**0.5**	**1**	**0.5**	223	** > 8**	> 8	> 8
	IMR		**0.25**	**0.5**	**0.25**		**0.25**	**0.25**	**0.25**		**2**	> 8	> 8		**0.25**	**0.25**	**0.25**		**8**	> 8	> 8
*Serratia marcescens*	IMI	4013	0.5	**2**	0.5	3523	0.5	1	0.5	490	2	> 8	2	3840	0.5	1	0.5	173	**8**	> 8	> 8
	IMR		0.5	**1**	0.5		0.5	1	0.5		2	> 8	2		0.5	1	0.5		**4**	> 8	> 8
*Klebsiella aerogenes*	IMI	2653	**1**	**2**	**1**	2030	**0.5**	**1**	**1**	623	**2**	**8**	**2**	2551	**1**	**2**	**1**	102	**8**	> 8	**4**
	IMR		**0.25**	**0.5**	**0.25**		**0.25**	**0.5**	** ≤ 0.12**		**0.5**	**1**	**0.5**		**0.25**	**0.5**	**0.25**		**0.5**	> 8	**0.5**
*Citrobacter freundii*	IMI	1553	**0.5**	**2**	**1**	1384	**0.5**	**1**	**1**	169	**2**	> 8	**2**	1489	**0.5**	**1**	**1**	64	**8**	> 8	**4**
	IMR		**0.25**	**0.5**	**0.25**		**0.25**	**0.25**	** ≤ 0.12**		**0.5**	> 8	**0.25**		**0.25**	**0.25**	**0.25**		**4**	> 8	** > 8**
Chromosomal AmpC nonproducers	IMI	78,766	**0.25**	**1**	≤ 0.12	72,907	≤ 0.12	**0.5**	≤ 0.12	5859	** > 8**	> 8	> 8	73,737	≤ 0.12	**0.5**	≤ 0.12	5029	** > 8**	> 8	> 8
	IMR		** ≤ 0.12**	**0.5**	≤ 0.12		≤ 0.12	**0.25**	≤ 0.12		**2**	> 8	> 8		≤ 0.12	**0.25**	≤ 0.12		**4**	> 8	> 8
*Escherichia coli*	IMI	46,649	≤ 0.12	0.25	≤ 0.12	45,843	≤ 0.12	0.25	≤ 0.12	806	** > 8**	> 8	> 8	46,004	≤ 0.12	**0.25**	≤ 0.12	645	> 8	> 8	> 8
	IMR		≤ 0.12	0.25	≤ 0.12		≤ 0.12	0.25	≤ 0.12		**8**	> 8	> 8		≤ 0.12	0.25	≤ 0.12		> 8	> 8	> 8
*Klebsiella pneumoniae*	IMI	27,423	0.25	** > 8**	**0.25**	22,476	0.25	0.5	**0.25**	4947	** > 8**	> 8	> 8	23,125	0.25	0.25	**0.25**	4298	** > 8**	> 8	> 8
	IMR		0.25	**1**	** ≤ 0.12**		0.25	0.5	** ≤ 0.12**		**2**	> 8	> 8		0.25	0.25	** ≤ 0.12**		**4**	> 8	> 8
*Klebsiella oxytoca*	IMI	3329	0.25	0.5	0.25	3238	0.25	0.5	0.25	91	**8**	> 8	> 8	3253	0.25	**1**	0.25	76	** > 8**	> 8	> 8
	IMR		0.25	0.5	0.25		0.25	0.5	0.25		**2**	> 8	> 8		0.25	**0.5**	0.25		**8**	> 8	> 8
*Citrobacter koseri*	IMI	1365	≤ 0.12	0.25	≤ 0.12	1350	≤ 0.12	0.25	≤ 0.12	15	**4**	> 8	ND	1355	≤ 0.12	0.25	≤ 0.12	10	**8**	> 8	ND
	IMR		≤ 0.12	0.25	≤ 0.12		≤ 0.12	0.25	≤ 0.12		**2**	> 8	ND		≤ 0.12	0.25	≤ 0.12		**4**	> 8	ND

*AmpC* Ambler class C β-lactamase, *CLSI* Clinical and Laboratory Standards Institute, *EUCAST* European Committee on Antimicrobial Susceptibility Testing, *IMI* Imipenem, *IMR* imipenem/relebactam, *MIC* Minimum inhibitory concentration, *ND* Not detected, *NS* nonsusceptible, *S* Susceptible

^a^Isolates were collected between 2018 and 2020

Bolded MIC values represent MIC_50/90_ and mode MIC values that were affected by the addition of relebactam to imipenem

*N* is the number of isolates classified as S or NS for each bacteria species

Among all Enterobacterales isolates (*N* = 91,769), the addition of relebactam increased imipenem susceptibility from 91.8% to 95.8% (see Additional file 2A) and reduced the MIC_50/90_ from 0.25/1 to ≤ 0.12/0.5 µg/mL (Table 1). For the 7493 isolates of Enterobacterales classified as imipenem-NS, the addition of relebactam restored imipenem susceptibility in 3704 (49.4%) isolates (see Additional file 2B) and reduced the MIC_50_ from > 8 to 2 µg/mL (Table 1). Among molecularly characterized isolates for which relebactam restored imipenem susceptibility, a majority (52.4%) encoded *K. pneumoniae* carbapenemases (KPCs), whereas MBLs (53.6%) and oxacillinase (OXA)-48 family β-lactamases (44.0%) were common among imipenem-NS isolates for which relebactam did not restore imipenem susceptibility. In addition, relebactam enhanced imipenem susceptibility among isolates of Enterobacterales classified as imipenem-S (*N* = 84,276) by inducing a shift toward lower MIC values (see Additional file 2C), with the MIC_50_ being reduced from 0.25 to ≤ 0.12 µg/mL (Table 1).

**Fig. 2:**
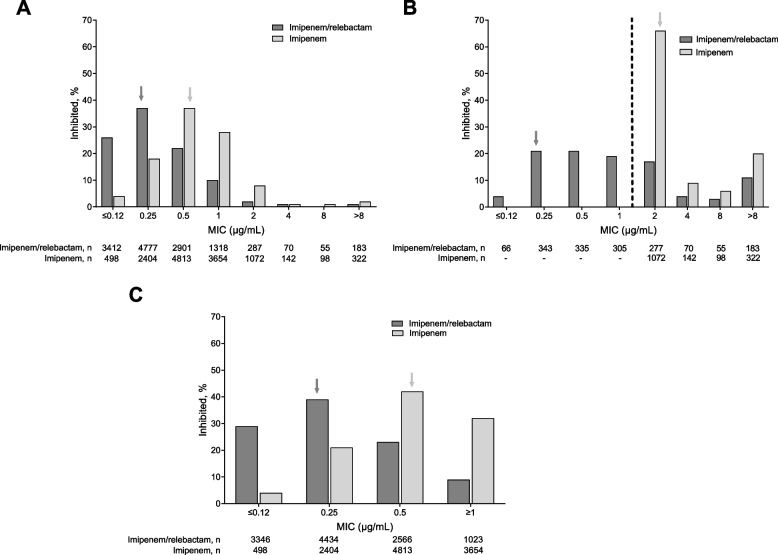
Relebactam restores the activity of imipenem in chromosomal AmpC–producing Enterobacterales isolates. **A** all (*N* = 13,003). **B** imipenem-NS (*N* = 1634). **C** imipenem-S (*N* = 11,369). Percentage represents *n*/*N* × 100%, where *n* was the number of isolates meeting the MIC threshold and *N* was the total number of isolates based on the CLSI 2021 clinical breakpoints for imipenem and imipenem/relebactam (both MIC ≤ 1 μg/mL for susceptibility) and subsequently categorized as either S (MIC ≤ 1 μg/mL) or NS (MIC > 1 μg/mL) [18]. The dashed line indicate**s** the CLSI 2021 imipenem and imipenem/relebactam susceptibility breakpoints. The arrows indicate mode MIC values. Enterobacterales chromosomal AmpC**–**producing species included *Enterobacter cloacae, Serratia marcescens, Klebsiella aerogenes,* and *Citrobacter freundii*. AmpC, Ambler class C β-lactamase; CLSI, Clinical and Laboratory Standards Institute; MIC, minimum inhibitory concentration; NS, nonsusceptible; S, susceptible

Because AmpC-producing Enterobacterales species have reduced susceptibility to imipenem [2, 19] and imipenem is a potent inducer of AmpC expression [4], we further analysed Enterobacterales species as separate subgroups based upon their capacity for chromosomal AmpC production. The chromosomal AmpC producers were *E. cloacae*, *S. marcescens*, *K. aerogenes*, and *C. freundii*; the nonproducers were *E. coli*, *K. pneumoniae*, *K. oxytoca,* and *C. koseri.* Among all isolates from chromosomal AmpC–producing species (*n* = 13,003), the addition of relebactam increased imipenem susceptibility from 87.4% to 95.4% (Fig. 2A) and reduced the MIC_50/90_ from 0.5/2 to 0.25/1 µg/mL; in addition, the mode MIC was reduced from 0.5 to 0.25 µg/mL (Table 1). Among imipenem-NS isolates from chromosomal AmpC–producing species (*n* = 1634), the addition of relebactam restored susceptibility to 64.2% of isolates (Fig. 2B), reduced the MIC_50_ from 2 to 1 µg/mL, and the mode MIC from 2 to 0.25 µg/mL (Table 1). Molecular characterization of imipenem-NS isolates for which relebactam restored imipenem susceptibility found that carbapenemases were present in 16.7% of isolates, with KPC as the most common carbapenemase, present in 13.7% of isolates (see Additional file 1). MBLs were present in 59.4% of imipenem-NS isolates for which relebactam did not restore imipenem susceptibility (see Additional file 1). With regards to imipenem-S isolates from chromosomal AmpC–producing species (*n* = 11,369), the addition of relebactam caused a shift towards reduced MICs (Fig. 2C) and reduced the MIC_50/90_ from 0.5/1 to 0.25/0.5 µg/mL; in addition, the mode MIC was reduced from 0.5 to 0.25 µg/mL (Table 1).

**Fig. 3:**
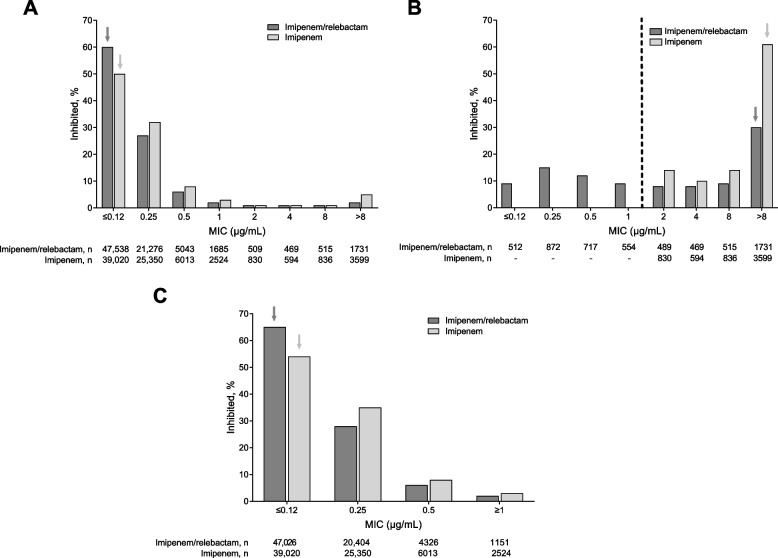
Relebactam enhances the activity of imipenem among chromosomal AmpC–nonproducing Enterobacterales isolates. **A** all (*N* = 78,766). **B** imipenem-NS (*N* = 5859). **C** imipenem-S (*N* = 72,907). Percentage represents *n*/*N* × 100%, where *n* was the number of isolates meeting the MIC threshold and *N* was the total number of isolates based on the CLSI 2021 clinical breakpoints for imipenem and imipenem/relebactam (both MIC ≤ 1 μg/mL for susceptibility) and subsequently categorized as either S (MIC ≤ 1 μg/mL) or NS (MIC > 1 μg/mL) [18]. The dashed line indicate**s** the CLSI 2021 imipenem and imipenem/relebactam susceptibility breakpoints. The arrows indicate mode MIC values. Enterobacterales chromosomal AmpC**–**nonproducing species included *Escherichia coli, Klebsiella pneumoniae* and *Klebsiella oxytoca,* and *Citrobacter koseri*. AmpC, Ambler class C β-lactamase; CLSI, Clinical and Laboratory Standards Institute; MIC, minimum inhibitory concentration; NS, nonsusceptible; S, susceptible

Among all Enterobacterales isolates from chromosomal AmpC–nonproducing species (*N* = 78,766) (Fig. 3A), the addition of relebactam increased imipenem susceptibility from 92.6% to 95.9% and reduced the MIC_50/90_ from 0.25/1 to ≤ 0.12/0.5 µg/mL (Table 1). Among imipenem-NS isolates from chromosomal AmpC–nonproducing species (*n* = 5859), the addition of relebactam restored susceptibility to 45.3% of isolates (Fig. 3B) and reduced the MIC_50_ from > 8 to 2 µg/mL (Table 1). Molecular characterization of these isolates found that KPC was present in 68.1% of isolates for which relebactam restored imipenem susceptibility; among isolates for which relebactam did not restore imipenem susceptibility, 36.1% encoded an OXA-48 family β-lactamase, 40.0% encoded an MBL, and 11.8% encoded both an OXA-48 family β-lactamase and an MBL (see Additional file 1). Among imipenem-S isolates from chromosomal AmpC–nonproducing species (*n* = 72,907), the addition of relebactam resulted in a small downward shift in MICs (Fig. 3C) and reduced the MIC_90_ from 0.5 to 0.25 µg/mL (Table 1).

The MIC data were also evaluated using imipenem European Committee on Antimicrobial Susceptibility Testing (EUCAST) susceptibility breakpoints of for Enterobacterales and 4 µg/mL for *P. aeruginosa* and imipenem/relebactam susceptibility breakpoints of 2 µg/mL for both Enterobacterales and *P. aeruginosa* (Table 1). The summary statistics using either CLSI or EUCAST breakpoints were largely similar for *P. aeruginosa* and Enterobacterales; they were either identical or within a single MIC dilution regardless of which interpretive criteria were applied. One notable difference was that for imipenem nonsusceptible isolates from chromosomal AmpC-producing Enterobacterales species, the imipenem mode MIC increased from 2 µg/mL using CLSI criteria to > 8 µg/mL using EUCAST criteria, as those isolates with MICs of 2 µg/mL were categorized as susceptible. Although there was no longer a reduction in the imipenem mode MIC in the presence of relebactam for these isolates, the MIC_50_ was still reduced by at least two dilutions, from > 8 to 4 µg/mL.

We evaluated the imipenem/relebactam susceptibility of imipenem-nonsusceptible isolates by region because of the geographic variation in the prevalence of carbapenemase enzymes among Enterobacterales inhibited by relebactam (*i.e.*, KPC) and those not inhibited by relebactam (*i.e.*, MBLs and OXA-48 family β-lactamases)[20], (Additional file 3). Relebactam restored imipenem susceptibility to > 70% of imipenem-nonsusceptible Enterobacterales isolated from Latin America, North America, or the South Pacific, where isolates frequently encode KPC or are carbapenemase negative. In Asia and Europe, where OXA-48 family β-lactamases and MBLs, respectively, are more common, relebactam restored imipenem susceptibility to 42.0% and 45.5%, respectively, of imipenem-NS Enterobacterales isolates. In addition, relebactam restored imipenem susceptibility to 63.7% to %-75.1% of imipenem-NS *P. aeruginosa* isolates from Asia, Europe, the Middle East, and North America, as well as 91.9% of isolates from the South Pacific. The rate of imipenem/relebactam susceptibility among imipenem-NS *P. aeruginosa* isolates was lower for isolates from Africa (48.3%) and Latin America (52.2%).

## Discussion

The present study expanded analysis of previous findings demonstrating that relebactam restores imipenem activity in imipenem-NS Enterobacterales and *P. aeruginosa* isolates and enhances imipenem activity in imipenem-S *P*. *aeruginosa* isolates [16]. In addition, this study extended the analysis of relebactam with imipenem among Enterobacterales species and evaluated Enterobacterales species as a function of their capacity to produce chromosomally encoded AmpC.

Among imipenem-NS isolates, relebactam restored susceptibility in chromosomal AmpC–nonproducing species (*e.g.*, *K. pneumoniae*), largely by inhibition of KPC, and in chromosomal AmpC–producing species (*e.g.*, *E. cloacae*), presumably through inhibition of the chromosomal AmpC enzyme. Relebactam restored imipenem susceptibility to approximately two-thirds and one-half of chromosomal AmpC–producing and AmpC–nonproducing Enterobacterales species, respectively, demonstrating that relebactam-mediated inhibition of β-lactamase activity can prevent loss of carbapenem susceptibility. Notable exceptions to the decreases in MIC values observed with imipenem/relebactam occurred in isolates encoding certain β-lactamases (*i.e.*, MBL and OXA). These isolates were minimally affected or unaffected by relebactam, which is consistent with lack of inhibition of class B and class D β-lactamases [14]. Collectively, these observations support previous findings that relebactam at a concentration of 4 µg/mL lowered imipenem MIC values [2, 16].

**Fig. 4:**
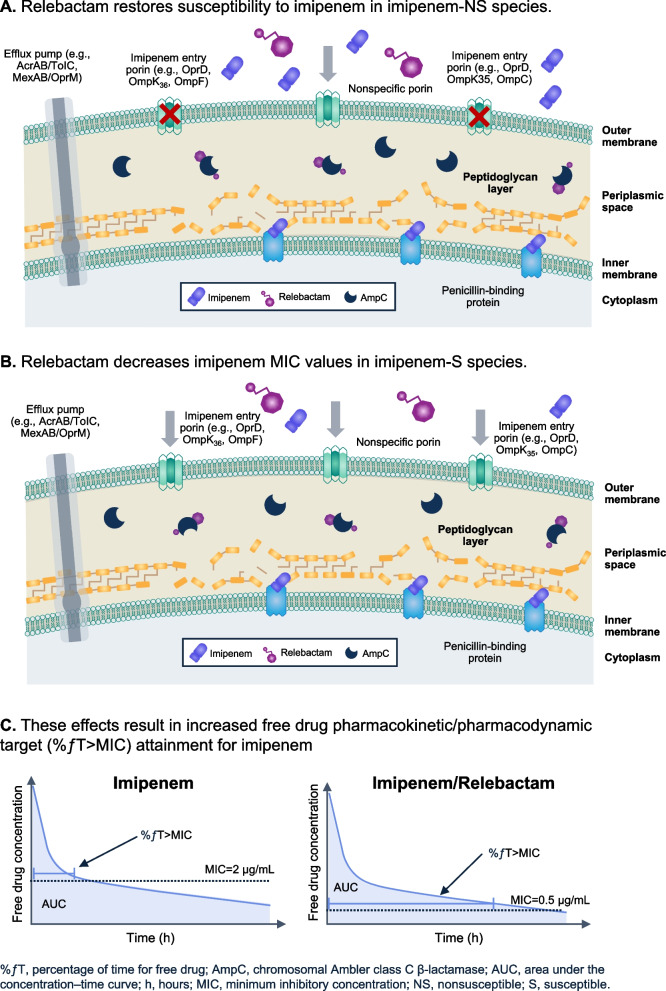
Effect of relebactam on susceptibility of *Pseudomonas aeruginosa* and Enterobacterales species to imipenem. **A** Relebactam restores susceptibility to imipenem in imipenem-NS *Pseudomonas aeruginosa* and Enterobacterales species. **B** Relebactam decreases imipenem MIC values in imipenem-S species. **C** These effects result in increased free drug pharmacokinetic/pharmacodynamic target (%ƒT > MIC) attainment for imipenem. %ƒT, percentage of time of free drug; AmpC, Ambler class C β-lactamase; AUC, area under the concentration–time curve; MIC, minimum inhibitory concentration; NS, nonsusceptible; S, susceptible

The mechanism of action for relebactam is inhibition of Class A or Class C β-lactamases to facilitate restoration or enhancement of imipenem susceptibility in gram-negative bacteria (Fig. 4; Additional file 4). Imipenem enters the periplasm through outer membrane porins [21]. Figure 4A depicts the effects upon addition of relebactam to imipenem among imipenem-NS *P. aeruginosa* and Enterobacterales isolates. In the absence of acquired carbapenemases (*e.g.*, KPC, MBL, etc.), imipenem nonsusceptibility among *P. aeruginosa* and Enterobacterales is due to two factors: 1) loss of the imipenem entry porins (*e.g.*, OprD, OmpK_36_, OmpF), which reduces entry of imipenem into the periplasm and 2) induced expression of the chromosomally encoded AmpC β-lactamase, which, although an inefficient carbapenemase, can degrade this reduced concentration of imipenem. Imipenem is a potent inducer of AmpC β-lactamases; therefore, whenever imipenem is present in a patient or an in vitro assay, AmpC will be hyperproduced [14, 21]. Relebactam likely restores imipenem susceptibility to these isolates by inhibiting AmpC, thereby allowing imipenem, which has entered the cell through nonspecific porins, to reach the target penicillin-binding proteins and exert its antibacterial effect, as observed for *P. aeruginosa* [16]. Among surveillance and genetically modified isolates, it is important to note that neither imipenem nor relebactam are substrates of efflux pumps [16, 22].

In contrast to imipenem-NS isolates, imipenem access to the periplasmic space through outer membrane porins (*e.g.*, OprD, OmpK_36_, OmpF) is efficient in imipenem-S isolates, and the greater concentration of imipenem in the periplasm results in an antibacterial effect, even in the presence of induced AmpC (Fig. 4B) [21]. Chromosomal AmpC expression leads to an increase in imipenem MIC values without resulting in loss of susceptibility. In the presence of relebactam, the slow hydrolysis of imipenem by chromosomal AmpC is impeded and the MIC values of imipenem are thereby lowered, enhancing imipenem susceptibility [16].

The clinical implications of this lowered imipenem/relebactam MIC, compared with imipenem alone for both *P. aeruginosa* and the Enterobacterales are informative for an evaluation of adequacy of dose from a pharmacokinetic (PK)/pharmacodynamic (PD) perspective. The importance of achieving established PD targets with standard dosing regimens of older β-lactams has been heightened in recent years, given reports of underdosing of these β-lactam agents, particularly among critically ill patients with ARC [7, 8]. Concerns regarding underdosing have led to multiple changes that have had implications in routine clinical practice, such as lowering of MIC susceptibility breakpoints as a method to ensure higher doses are administered [23] and recommendations to administer prolonged or continuous infusions of β-lactams to increase the probability that adequate PD exposures are achieved [24, 25]. With this context in mind, the established PK/PD target for imipenem, and all carbapenems, is the percentage of time the free drug concentration remains above the MIC of the infecting organism (%*f*T > MIC), with maximum bactericidal effect achieved at an %ƒT > MIC of 30%–40% [26–29]. For the β-lactamase inhibitor relebactam, a ratio of the area under the unbound concentration–time curve to the MIC (*f*AUC/MIC) of 7.5 was associated with two-log kill in preclinical models [28–31]. High (> 90%) joint probability of target attainment for both imipenem and relebactam has been described for the 1.25-g IMI/REL dose (500 mg imipenem/500 mg cilastatin/250 mg relebactam) at an imipenem/relebactam MIC breakpoint of ≤ 2 µg/mL, which covers *P*. *aeruginosa* and Enterobacterales [24, 25]. Because the MIC is in the denominator of each PK/PD target (*e.g.*, *f*T > MIC and *f*AUC/MIC), the addition of relebactam as a strategy to lower the imipenem MIC is expected to result in higher PD exposures (Fig. 4C) and serves as an alternative approach to extending the infusion to attain higher PD exposures. Although purely speculative, higher exposures achieved upon the addition of relebactam to imipenem may have contributed to the efficacy observed in the RESTORE-IMI 1 and 2 studies [10, 12, 14, 32, 33]. Of particular importance are the similar 28-day all-cause mortality and favorable clinical and microbiologic response rates among patients with normal renal function compared with those with ARC (creatinine clearance ≥ 150 mL/min) among participants with hospital-acquired/ventilator-associated bacterial pneumonia and ARC from the RESTORE-IMI 2 study [13].

In this study, molecularly characterized imipenem-nonsusceptible Enterobacterales isolates that remained nonsusceptible in the presence of relebactam frequently encoded an OXA-48 family β-lactamase, illustrating the lack of activity of relebactam against these enzymes. Among the few isolates in which relebactam restored susceptibility, the vast majority (84%) had MICs of , interpreted as intermediate susceptibility by CLSI and susceptible by EUCAST, which was attributable to the weak carbapenemase activity of OXA-48 family β-lactamases [34]. Imipenem-susceptible isolates were not characterized in this study; however, Enterobacterales isolates encoding OXA-48 family β-lactamases are frequently susceptible to meropenem [35], suggesting these enzymes are likely present in imipenem-susceptible isolates as well. Attributing the intermediate imipenem susceptibility phenotype to the presence of an OXA-48 family β-lactamase in a particular isolate is challenging; MIC values frequently vary by a single dilution in testing, and the presence of additional β-lactamases and resistance mechanisms (*e.g.*, outer membrane porin loss) may be contributing factors. Overall, the results of this study reinforce prior findings that relebactam is an inhibitor class A/C β-lactamase and can restore imipenem susceptibility to isolates encoding these enzymes; correspondingly, the lack of relebactam activity toward class B/D β-lactamases is illustrated by the lack of meaningful restoration of imipenem susceptibility to isolates encoding these enzymes. From a clinical perspective, PK/PD data indicate that IMI/REL achieves high probability of target attainment for isolates with imipenem/relebactam MICs ≤ 2 µg/mL (*i.e.*, the EUCAST susceptible, standard-dosing regimen breakpoint) [33], and limited clinical data indicate favorable clinical and microbiologic outcomes in a small number of trial participants (n = 3) with imipenem/relebactam-susceptible isolates encoding OXA-48 family β-lactamases (unpublished data) [12].

A limitation of the study was the range of MICs assessed. Wider ranges may have allowed detection of larger modal shifts (*e.g.*, among imipenem-NS Enterobacterales). The relationship between bacterial susceptibility and antibacterial agent is complex; therefore, MIC may not be the best indicator of effectiveness of a particular antibacterial agent [36]. In addition, we did not directly measure AmpC production, but it is well recognized that this is one of the primary mechanisms of resistance of *P. aeruginosa* and certain Enterobacterales species [19, 24, 37] and that relebactam inhibition of chromosomally encoded AmpC enzymes is responsible for reduction in imipenem MIC values when acquired β-lactamases are not present [16].

The results of the present study indicated that encoded *MBL* and *OXA* genes contributed to certain Enterobacterales isolates remaining imipenem-NS after the addition of relebactam; however, the possibility remains that unidentified resistance mechanisms within these isolates contributed to their phenotype. The characterisation needed to confirm the presence of other resistance pathways was beyond the scope of the present study. Furthermore, although imipenem/relebactam circumvents certain resistance mechanisms in vitro with the associated decrease in MIC described here, other patient-specific factors may impact effectiveness to a greater extent.

## Conclusions

Relebactam inhibits the ability of AmpC and KPC β-lactamases to hydrolyse imipenem in vitro, thereby restoring imipenem susceptibility among nonsusceptible isolates and enhancing imipenem susceptibility among susceptible isolates of *P. aeruginosa* and Enterobacterales. The reduction of imipenem modal MIC values with relebactam may result in a higher probability of target attainment in patients.

## Materials and methods

Between 2018 and 2020, 243 unique participating sites collected up to 250 consecutive isolates each of aerobic gram-negative bacteria per year for the SMART program. Participating sites were located in 219 cities across 60 countries. The following number of isolates were collected from adult patients (≥ 18 years of age) at each site, each year hospitalized with: cUTIs (*n* = 50), cIAIs (*n* = 50), lower respiratory tract infections (*n* = 100), or bloodstream infections (*n* = 50). One isolate per species per patient per year was included. After collection, isolates were submitted to a central laboratory (International Health Management Associates, Inc [IHMA], Schaumburg, Illinois, USA) for analysis. All methods were carried out in accordance with the ethical principles Declaration of Helsinki and all relevant guidelines and regulations. Ethical approval and informed consent were not required because all isolates received into the study followed multiple subcultures and were completely de-identified. The secondary research use of de-identified isolates is considered exempt research according to the Regulations for the Protection of Human Subjects in Research of the U.S. Department of Health and Human Services, Office for Human Research Protections (45 CFR 46).

Confirmation of *P. aeruginosa* and Enterobacterales isolate identity was performed by IHMA using matrix-assisted laser desorption ionization time-of-flight mass spectrometry (Bruker Daltonics, Billerica, Massachusetts, USA). Based on current IMI/REL indications, Enterobacterales species included in this analysis were *Escherichia coli, Klebsiella pneumoniae, Enterobacter cloacae, Serratia marcescens, Klebsiella aerogenes, Klebsiella oxytoca*, and *Citrobacter freundii*. In addition, *Citrobacter koseri* was also included because it is a carbapenem-resistant species that increasingly has been seen in hospital settings [38]. Isolates of *Enterobacter cloacae, Serratia marcescens, Klebsiella aerogenes,* and *Citrobacter freundii* were categorized as potential AmpC-producing species based on the presence of an AmpC-encoding gene and the potential for derepression due to genetic mutation or the presence of an inducing β-lactam antibacterial agent, such as imipenem [6].

Antibacterial susceptibility testing was performed at IHMA using CLSI standard broth microdilution methods [39]. The appropriate American Type Culture Collection control strains were used each day as quality-control measures in accordance with CLSI guidelines. Isolates were tested for susceptibility to both imipenem alone and imipenem/relebactam. Per CLSI recommendations, imipenem was diluted according to a two-fold gradient and tested in combination with a fixed concentration (4 μg/mL) of relebactam [18]. For *P. aeruginosa*, the CLSI breakpoints for imipenem and imipenem/relebactam susceptibility were both  [18]. The CLSI breakpoints for imipenem and imipenem/relebactam susceptibility were both ≤ 1 μg/mL for Enterobacterales [18]. Isolates with MIC values that exceeded these CLSI breakpoints were deemed nonsusceptible. The range of imipenem and imipenem/relebactam MICs tested for *P. aeruginosa* was ≤ 0.12 to > 32 µg/mL and for Enterobacterales was ≤ 0.12 to > 8 µg/mL.

In this study, isolates of *P. aeruginosa* and Enterobacterales classified as nonsusceptible to imipenem that were characterized molecularly for gene-encoded β-lactamases using previously described multiplex polymerase chain reaction assays and full-gene DNA sequencing techniques [40, 41] were evaluated. Over the current study period (2018–2020), 75% of imipenem-NS isolates from the species evaluated were analysed. Screening included assessment for gene-encoded MBLs, including imipenemase, Verona integron-encoded metallo-β-lactamase, New Delhi metallo-β-lactamase, and São Paulo MBL; serine β-lactamases (KPC; OXA) and chromosomally encoded PDC. The number of imipenem-nonsusceptible isolates molecularly characterized from the evaluated species is provided in Additional file 1. Isolates in which no carbapenemase-encoding genes were identified were characterized as carbapenemase negative. Gene-flanking primers were used to amplify and sequence (Sanger) all detected genes encoding carbapenemases and PDC for all Enterobacterales isolates in the study and for *P. aeruginosa* isolates from 2018 to 2019. *P. aeruginosa* isolates collected in 2020 that met the screening criteria were characterized by short-read whole-genome sequencing (Illumina HiSeq 2 × 150 base-pair reads) to a targeted coverage depth of 100 × [42] and analyzed using the CLC Genomics Workbench (Qiagen, Germantown, Maryland, USA). The ResFinder database was used to detect β-lactamase genes [43].

## Supplementary Information

**Additional file 1.** Molecular characterization of resistance mechanisms on the subset of samples with data available.

**Additional file 2.** Relebactam enhances the activity of imipenem among Enterobacterales.

**Additional file 3.** Imipenem/relebactam susceptibility of imipenem nonsusceptible isolates by region.

**Additional file 4.**  Graphical abstract.

## Data Availability

Datasets used and analyzed for this study are available from the corresponding author upon reasonable request.
