# Supplementary material for: Relebactam restores susceptibility of resistant Pseudomonas aeruginosa and Enterobacterales and enhances imipenem activity against chromosomal AmpC-producing species: analysis of global SMART 2018–2020
Source: BMC Microbiol. 2023 Jun 13;23:165. doi: 10.1186/s12866-023-02864-3 (PMC10262423; doi:10.1186/s12866-023-02864-3)
Supplement: Supplementary file 1 — Additional file 1. Molecular characterization of resistance mechanisms on the subset of samples with data available. [file 12866_2023_2864_MOESM1_ESM.pdf]

Additional File 1 Molecular characterization of resistance mechanisms on the subset of samples with data available.

			IMR-S														IMR-NS													
				IMR-S Isolates (% of IMI-NS Isolates)														IMR-NS Isolates (% of Characterized IMI-NS Isolates)												
Organism	All IMI-NS Isolates	Characterized IMI-NS Isolates (% of All IMI-NS Isolates)	IMR-S Isolates (% of Characterized IMI-NS Isolates)	CP^+	KPC	OXA	MBL	GES	GPC	KPC + MBL	KPC + OXA	MBL + GES	MBL + OXA	OXA + GES	MBL + KPC + GES	CP^-	IMR-NS Isolates (% of Characterized IMI-NS Isolates)	CP^+	KPC	OXA	MBL	GES	GPC	KPC + MBL	KPC + OXA	MBL + GES	MBL + OXA	OXA + GES	MBL + KPC + GES	CP^-
Pseudomonas aeruginosa	8356	6543 (78.3)	4192 (64.1)	10 (0.2)	2 (0.1)	0 (0)	6 (0.1)	2 (0.1)	0 (0)	0 (0)	0 (0)	0 (0)	0 (0)	0 (0)	0 (0)	4182 (99.8)	2351 (35.9)	1044 (44.4)	60 (2.6)	0 (0)	804 (34.2)	105 (4.5)	1 (0.04)	31 (1.3)	0 (0)	43 (1.8)	0 (0)	0 (0)	0 (0)	1307 (55.6)
Enterobacterales	7493	5306 (70.8)	2733 (51.5)	1723 (63.0)	1420 (52.0)	280 (10.3)	10 (0.4)	0 (0)	0 (0)	8 (0.3)	4 (0.2)	0 (0)	1 (0.04)	0 (0)	0 (0)	1010 (37.0)	2573 (48.5)	2327 (90.4)	95 (3.7)	847 (32.9)	1044 (40.6)	2 (0.1)	0 (0)	37 (1.4)	3 (0.1)	16 (0.6)	280 (10.9)	1 (0.04)	2 (0.1)	246 (9.6)
Chromosomal AmpC Producers	1634	1103 (67.5)	795 (72.1)	133 (16.7)	101 (12.7)	19 (2.4)	5 (0.6)	0 (0)	0 (0)	7 (0.9)	1 (0.1)	0 (0)	0 (0)	0 (0)	0 (0)	662 (83.3)	308 (27.9)	231 (75.0)	17 (5.5)	30 (9.7)	161 (52.3)	0 (0)	0 (0)	10 (3.3)	0 (0)	0 (0)	12 (3.9)	1 (0.3)	0 (0)	77 (25.0)
Enterobacter cloacae	352	280 (79.6)	145 (51.8)	63 (43.5)	44 (30.3)	9 (6.2)	4 (2.8)	0 (0)	0 (0)	6 (4.1)	0 (0)	0 (0)	0 (0)	0 (0)	0 (0)	82 (56.6)	135 (48.2)	125 (92.6)	4 (3.0)	6 (4.4)	106 (78.5)	0 (0)	0 (0)	3 (2.2)	0 (0)	0 (0)	6 (4.4)	0 (0)	0 (0)	10 (7.4)
Serratia marcescens	490	139 (28.4)	48 (34.5)	35 (72.9)	35 (72.9)	0 (0)	0 (0)	0 (0)	0 (0)	0 (0)	0 (0)	0 (0)	0 (0)	0 (0)	0 (0)	13 (27.1)	91 (65.5)	52 (57.1)	10 (11.0)	9 (9.9)	26 (28.6)	0 (0)	0 (0)	5 (5.5)	0 (0)	0 (0)	1 (1.1)	1 (1.1)	0 (0)	39 (42.9)
Klebsiella aerogenes	623	535 (85.9)	496 (92.7)	13 (2.6)	6 (1.2)	6 (1.2)	1 (0.2)	0 (0)	0 (0)	0 (0)	0 (0)	0 (0)	0 (0)	0 (0)	0 (0)	483 (97.4)	39 (7.3)	16 (41.0)	3 (7.7)	3 (7.7)	9 (23.1)	0 (0)	0 (0)	0 (0)	0 (0)	0 (0)	1 (2.6)	0 (0)	0 (0)	23 (59.0)
Citrobacter freundii	169	149 (88.2)	106 (71.1)	22 (20.8)	16 (15.1)	4 (3.8)	0 (0)	0 (0)	0 (0)	1 (0.9)	1 (0.9)	0 (0)	0 (0)	0 (0)	0 (0)	84 (79.3)	43 (28.9)	38 (88.4)	0 (0)	12 (27.9)	20 (46.5)	0 (0)	0 (0)	2 (4.7)	0 (0)	0 (0)	4 (9.3)	0 (0)	0 (0)	5 (11.6)
Chromosomal AmpC Non-Producers	5859	4203 (71.7)	1938 (46.1)	1590 (82.0)	1319 (68.1)	261 (13.5)	5 (0.3)	0 (0)	0 (0)	1 (0.1)	3 (0.2)	0 (0)	1 (0.1)	0 (0)	0 (0)	348 (18.0)	2265 (53.9)	2096 (92.5)	78 (3.4)	817 (36.1)	883 (39.0)	2 (0.1)	0 (0)	27 (1.2)	3 (0.1)	16 (0.7)	268 (11.8)	0 (0)	2 (0.1)	169 (7.5)
Escherichia coli	806	469 (58.2)	161 (34.3)	100 (62.1)	58 (36.0)	40 (24.8)	2 (1.2)	0 (0)	0 (0)	0 (0)	0 (0)	0 (0)	0 (0)	0 (0)	0 (0)	61 (37.9)	308 (65.7)	266 (86.4)	0 (0)	47 (15.3)	193 (62.7)	0 (0)	0 (0)	3 (1.0)	0 (0)	4 (1.3)	19 (6.2)	0 (0)	0 (0)	42 (13.6)
Klebsiella pneumoniae	4947	3654 (73.9)	1740 (47.6)	1461 (84.0)	1236 (71.0)	218 (12.5)	2 (0.1)	0 (0)	0 (0)	1 (0.1)	3 (0.2)	0 (0)	1 (0.1)	0 (0)	0 (0)	279 (16.0)	1914 (52.4)	1789 (93.5)	78 (4.1)	762 (39.8)	659 (34.4)	1 (0.1)	0 (0)	24 (1.3)	3 (0.2)	11 (0.6)	249 (13.0)	0 (0)	2 (0.1)	125 (6.5)
Klebsiella oxytoca	91	70 (76.9)	31 (44.3)	26 (83.9)	25 (80.7)	0 (0)	1 (3.2)	0 (0)	0 (0)	0 (0)	0 (0)	0 (0)	0 (0)	0 (0)	0 (0)	5 (16.1)	39 (55.7)	37 (94.9)	0 (0)	6 (15.4)	29 (74.4)	1 (2.6)	0 (0)	0 (0)	0 (0)	1 (2.6)	0 (0)	0 (0)	0 (0)	2 (5.1)
Citrobacter koseri	15	10 (66.7)	6 (60.0)	3 (50.0)	0 (0)	3 (50.0)	0 (0)	0 (0)	0 (0)	0 (0)	0 (0)	0 (0)	0 (0)	0 (0)	0 (0)	3 (50.0)	4 (40.0)	4 (100.0)	0 (0)	2 (50.0)	2 (50.0)	0 (0)	0 (0)	0 (0)	0 (0)	0 (0)	0 (0)	0 (0)	0 (0)	0 (0)

AmpC, Ambler class C β-lactamase; CP, carbapenemase; GES, Guiana extended-spectrum β-lactamase; IMI, imipenem; IMP, imipenemase; IMR, imipenem/relebactam; KPC, *K. pneumoniae* carbapenemase; IMR, imipenem/relebactam; MBL, metallo-β-lactamase; NDM, New Delhi metallo-β-lactamase; NS, nonsusceptible; OXA, oxacillinase; REL, relebactam; S, susceptible; VIM, Verona integron-encoded metallo-β-lactamase.
