# Supplementary material for: Relebactam restores susceptibility of resistant Pseudomonas aeruginosa and Enterobacterales and enhances imipenem activity against chromosomal AmpC-producing species: analysis of global SMART 2018–2020
Source: BMC Microbiol. 2023 Jun 13;23:165. doi: 10.1186/s12866-023-02864-3 (PMC10262423; doi:10.1186/s12866-023-02864-3)
Supplement: Supplementary file 2 — Additional file 2. Relebactam enhances the activity of imipenem among Enterobacterales. [file 12866_2023_2864_MOESM2_ESM.pdf]

Additional File 2 Relebactam enhances the activity of imipenem among Enterobacterales isolates.

(**A**) all (*N* = 91,769).

(**B**) imipenem-NS (*N* = 7493).

(**C**) imipenem-S (*N* = 84,276).

A

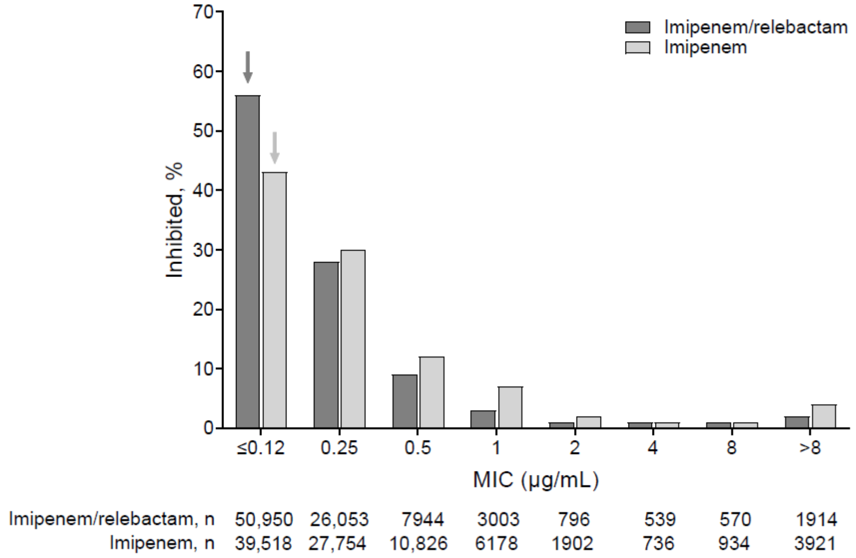

B

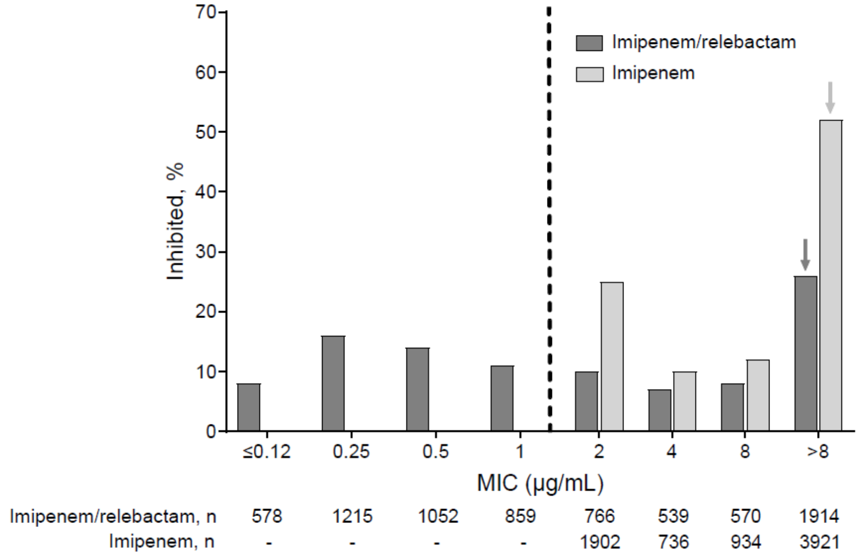

C

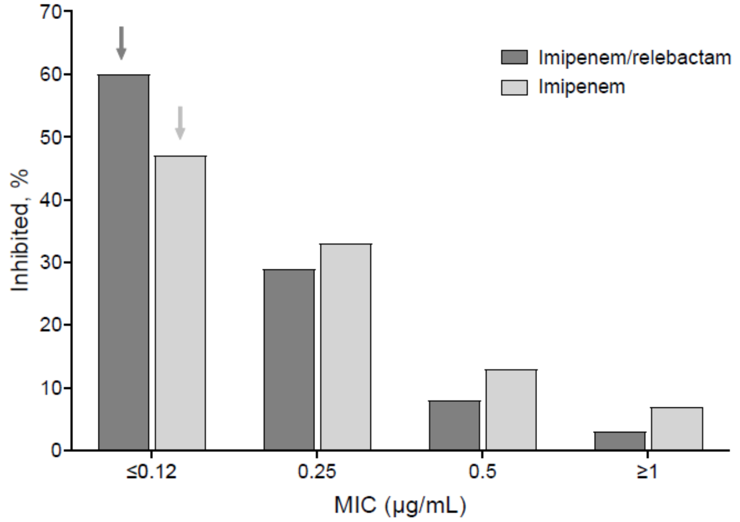

Imipenem/relebactam, n	50,372	24,838	6892	2174
Imipenem, n	39,518	27,754	10,826	6178

Percentage represents $n/N \times 100\%$, where n was the number of isolates meeting the MIC threshold and N was the total number of isolates based on the CLSI 2021 clinical breakpoints for imipenem and imipenem/relebactam (both MIC ≤1 µg/mL for susceptibility) and subsequently categorized as either S (MIC ≤1 µg/mL) or NS (MIC >1 µg/mL) [1]. The dashed line indicates the CLSI 2021 imipenem and imipenem/relebactam susceptibility breakpoints. The arrows indicate mode MIC values. Enterobacterales included *Escherichia coli, Klebsiella pneumoniae, Enterobacter cloacae, Serratia marcescens, Klebsiella oxytoca* and *Klebsiella aerogenes, and Citrobacter freundii* and *Citrobacter koseri.* CLSI, Clinical and Laboratory Standards Institute; MIC, minimum inhibitory concentration; NS, nonsusceptible; S, susceptible.

1. Clinical and Laboratory Standards Institute. Performance Standards for Antimicrobial Susceptibility Testing. 31st ed. CLSI supplement M100. Clinical Laboratory Standards Institute, Wayne, PA, 2021.
