# Supplementary material for: Relebactam restores susceptibility of resistant Pseudomonas aeruginosa and Enterobacterales and enhances imipenem activity against chromosomal AmpC-producing species: analysis of global SMART 2018–2020
Source: BMC Microbiol. 2023 Jun 13;23:165. doi: 10.1186/s12866-023-02864-3 (PMC10262423; doi:10.1186/s12866-023-02864-3)
Supplement: Supplementary file 3 — Additional file 3. Imipenem/relebactam susceptibility of imipenem nonsusceptible isolates by region. [file 12866_2023_2864_MOESM3_ESM.pdf]

Additional File 3. Imipenem/relebactam susceptibility of imipenem nonsusceptible isolates by region.

Organism	Region													
	Africa		Asia		Europe		Latin America		Middle East		North America		South Pacific	
	IMI-NS (*n*)	% IMR-S	IMI-NS (*n*)	% IMR-S	IMI-NS (*n*)	% IMR-S	IMI-NS (*n*)	% IMR-S	IMI-NS (*n*)	% IMR-S	IMI-NS (*n*)	% IMR-S	IMI-NS (*n*)	% IMR-S
Pseudomonas aeruginosa	415	48.3	2202	63.7	2352	65.2	1528	52.2	433	71.4	1193	75.1	233	91.9
Enterobacterales	528	17.1	2894	42.0	1655	45.5	1643	71.0	198	32.8	443	72.0	133	72.9
Chromosomal AmpC producers	56	35.7	433	61.2	266	53.0	413	68.5	28	71.4	329	71.4	109	78.0
Klebsiella aerogenes	9	88.9	167	91.0	93	92.5	113	94.2	15	86.7	161	91.9	58	98.3
Enterobacter cloacae	17	47.1	120	36.7	62	17.7	95	63.2	4	50.0	42	88.1	12	91.7
Citrobacter freundii	3	33.3	48	58.3	28	67.9	54	68.5	4	75.0	26	96.2	6	83.3
Serratia marcescens	27	11.1	98	41.8	83	30.1	73	50.7	5	40.0	100	25.0	33	36.4
Chromosomal AmpC nonproducers	472	14.8	2460	38.6	1389	44.1	1230	71.8	170	26.5	114	73.7	24	50.0
Klebsiella pneumoniae	422	15.2	1978	43.7	1256	45.1	783	73.7	124	25.8	86	68.6	18	50.0
Escherichia coli	44	13.6	451	17.1	107	35.5	137	56.9	43	30.2	19	89.5	5	40.0
Klebsiella oxytoca	6	16.7	25	20.0	21	23.8	28	75.0	2	0	9	88.9	0	ND
Citrobacter koseri	0	ND	6	33.3	5	60.0	2	50.0	1	0	0	ND	1	100.0

AmpC, Ambler class C β-lactamase; IMI, imipenem; IMR, imipenem/relebactam; ND, not detected; NS, nonsusceptible; S, susceptible.
