# Supplementary material for: Relebactam restores susceptibility of resistant Pseudomonas aeruginosa and Enterobacterales and enhances imipenem activity against chromosomal AmpC-producing species: analysis of global SMART 2018–2020
Source: BMC Microbiol. 2023 Jun 13;23:165. doi: 10.1186/s12866-023-02864-3 (PMC10262423; doi:10.1186/s12866-023-02864-3)
Supplement: Supplementary file 4 — Additional file 4. Graphical abstract. [file 12866_2023_2864_MOESM4_ESM.pdf]

Relebactam Increases Susceptibility to Imipenem: Analysis of Global SMART 2018–2020

Relebactam inhibits the ability of AmpC and KPC β-lactamases to hydrolyze imipenem *in vitro*, thereby restoring imipenem susceptibility among nonsusceptible isolates (left) and enhancing imipenem susceptibility among susceptible isolates (right) of *P. aeruginosa* and Enterobacterales.

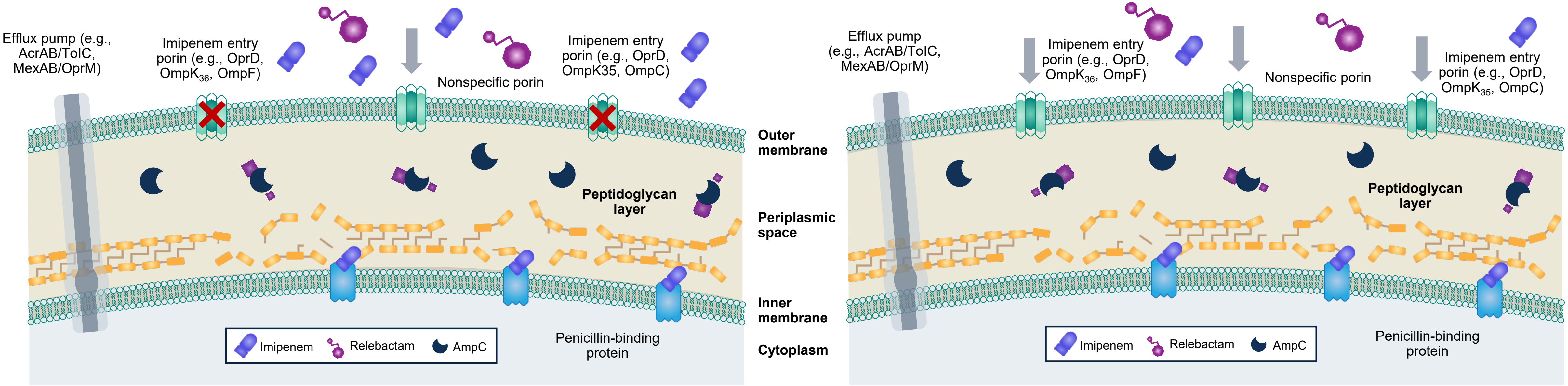

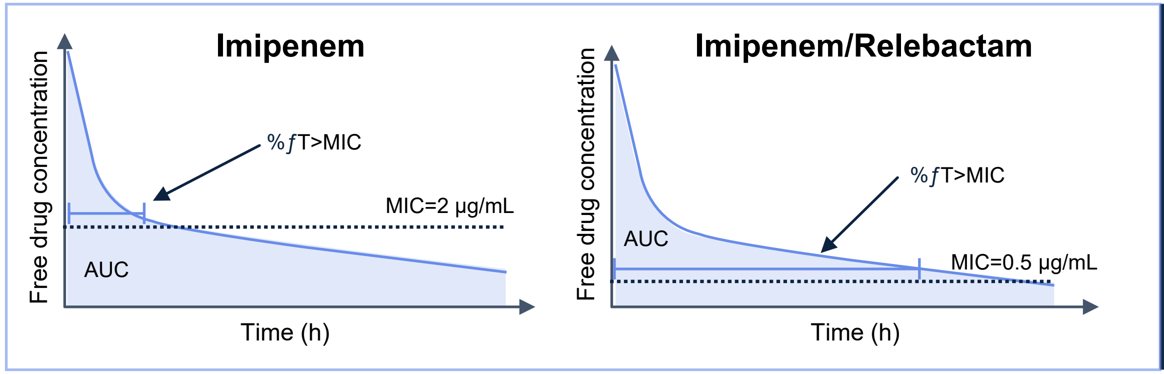

The lowering of imipenem MIC upon addition of relebactam may result in a higher probability of target attainment *in vivo*, further supporting the IMI/REL efficacy data observed in phase 3 clinical trials.

%ƒT, percentage of time for free drug; AmpC, chromosomal Ambler class C β-lactamase; AUC, area under the concentration–time curve; h, hours; KPC, *Klebsiella pneumoniae* carbapenemase; IMI/REL, imipenem/cilastatin/relebactam; MIC, minimum inhibitory concentration; SMART, Study for Monitoring Antimicrobial Resistance Trends.
